# 3,4-Dihydroxytoluene, a metabolite of rutin, inhibits inflammatory responses in lipopolysaccharide-activated macrophages by reducing the activation of NF-κB signaling

**DOI:** 10.1186/1472-6882-14-21

**Published:** 2014-01-13

**Authors:** Kang-Yi Su, Chao Yuan Yu, Ya-Ping Chen, Kuo-Feng Hua, Yi-Lin Sophia Chen

**Affiliations:** 1 Department of Biotechnology and Animal Science, National Ilan University, Shen-Lung Road, Ilan 260, Taiwan; 2 Department of Clinical Laboratory Sciences and Medical Biotechnology, College of Medicine, National Taiwan University, Taipei 100, Taiwan; 3 Department of Laboratory Medicine, National Taiwan University Hospital, Taipei 100, Taiwan

**Keywords:** *Saussurea involucrata*, Flavonoid, Anti-inflammation, Cyclooxygenase-2 (COX-2), Inducible nitric oxide synthase

## Abstract

**Background:**

*Saussurea involucrata* (Kar. et Kir.) (*S. involucrate*), is a rare traditional Chinese medicinal herb. Rutin and hispidulin as well as their metabolites are flavonoids of the flavonol type that abound in *S. involucrata*, which has been reported to inhibit nonoxidative advanced glycation end products which was involved in physiological inflammation. This study aims to investigate the role of 3,4-dihydroxytoluene (DHT), a metabolite of rutin, in inflammatory inhibition and its involved mechanism.

**Methods:**

This study utilized lipopolysaccharide (LPS) stimulated murine macrophage cell line RAW 264.7 as inflammatory model. The inhibitory effects of DHT were evaluated by the expression level of several inflammation markers such as inducible nitric oxide synthase (iNOS) and cyclooxygenase-2 (COX-2) in RAW264.7 after LPS treatment. In addition, underlying mechanisms, the activation of mitogen-activated protein kinases (MAPKs) and NF-κB, were also investigated.

**Results:**

Our results showed that DHT significantly suppressed the LPS-induced production of nitric oxide (NO), iNOS, and COX-2 in a dose-dependent manner without cytotoxicity. DHT also reduced the generation of proinflammatory cytokines majorly in tumor necrosis factor (TNF)-α and minor in interleukin (IL)-1β and IL-6. In addition, LPS-stimulated I-κBα phosphorylation and degradation followed by translocation of the nuclear factor κB (NF-kB)-p65 from the cytoplasm to the nucleus were attenuated after DHT treatment.

**Conclusions:**

Combined, the results suggest that DHT might exert anti-inflammatory effects in vitro in LPS stimulated RAW 264.7 macrophages and is potential in adjuvant treatment in inflammation disease.

## Background

*Saussurea involucrata* (Kar. et Kir.) (*S. involucrata*), known as the snow lotus, grows in the rocky habitats of mountains that reach or exceed an elevation of 2600 m in the Tian Shan and A’er Tai areas of China. In recent years, the wild population of *S. involucrata* has been facing the threat of depletion due to their particularly slow growth and excessively harvested from the wild for pharmaceutical preparations. *S. involucrata* is now nearly extinct and has been listed as a second grade national protected wild plant in China [1,2]. Based on the theories of traditional Chinese medicine, *S. involucrata* has the effect of warming the kidneys, activating *yang*, expelling wind, eliminating dampness, inducing menstruation, and promoting blood circulation. In folk medicine, it is used to treat rheumatoid arthritis, impotence, irregular menses, coughs and colds, stomachache, and altitude sickness, among other illnesses [3,4]. Rutin (3,3’,4’,5,7-pentahydroxyflavone-3-rhamnoglucoside) is a flavonoid of the flavonol type abundantly present in *S. involucrata.* It has been reported that rutin has several pharmacological properties, including antioxidant, anticarcinogenic, cytoprotective, antiplatelet, antithrombotic, vasoprotective, and cardioprotective properties [5-10] as well as neuroprotective [11]. In addition, it also has been demonstrated to have anticonvulsant properties in pentylenetetrazol models in rats and mice [12]. In the mention of antioxidative issue, our previous report showed rutin can ameliorated ischemic reperfusion injuries in the brain by reducing MDA level in plasma and elevating caspase-3 and PARP [13].

High oral doses of rutin (3,3’,4’,5,7-pentahydroxyflavone-3-rhamnoglucoside) protected diabetic rats against the formation of protein adducts in the skin [14]. Although the precise mechanism of this protection is not known, little or no dietary rutin is absorbed because gut microflora in the large intestine metabolize rutin to a variety of compounds that include quercetin and phenol derivatives such as 3,4-dihydroxyphenylacetic acid (DHPAA), 3,4-dihydroxytoluene (DHT), -hydroxyphenylacetic acid (HPAA), and 4-hydroxy-3-methoxyphenylacetic acid (homovanillic acid, HVA) [15-20]. Pashikanti et. al. reported that the rutin metabolites can efficiently and powerfully inhibit fluorescent and nonfluorescent belong to advanced glycation end products (AGEs) [21]. Since AGEs were an important source of reactive dicarbonyl species (RCS) involved in several pathophysiological conditions such as inflammation [22,23], we hypothesized that the metabolites can reduce inflammatory response. More importantly, rutin can reduce inflammatory cytokines such as IL-1 from the peritoneal macrophages (pMf). [17] Most of the rutin that is consumed is likely to be decomposed into low-molecular-weight phenolic acids by the colonic microflora [24,25]. However, it had not been investigated what kind of metabolites of rutin can have anti-inflammation effect. In addition, it was evidenced that most of the protective effects of flavonoids against oxidative stress in PC12 cells are continued despite biodegradation of the parent flavonoids. Taken together, this background has led us to examine the suppression of the inflammation effects of dietary supplements using rutin’s derives, such as DHPAA, DHT, HPAA, and HVA on LPS-induced inflammation in macrophage cells.

In this study, we utilized Lipopolysaccharide (LPS)-stimulated murine macrophage cell line RAW 264.7 as inflammatory model to test the effects of rutin derives. LPS can trigger groups of different toll-like receptors (TLR) especially TLR4 expressed on the cell surface of immune cells [26]. The binding of LPS to TLR4 triggers down-stream signaling cascades, including mitogen-activated protein kinases (MAPKs) [27] and the nuclear transcription factor kappa-B (NF-B) pathways [28], which lead to the production of inflammatory mediators from macrophages, such as tumor necrosis factor-alpha (TNF-α), interleukin-1 (IL-1), interleukin-6 (IL-6), and nitric oxide (NO) [29]. Our results indicated DHT, the phenol derivative of rutin, inhibited the NO, TNF-α in LPS-stimulated macrophages through I*κ*B-NF-*κ*B signaling rather than MAPK, ERK1/2, p38 and JNK1/2 pathways. These results provided a model of DHT for anti-inflammation usage.

## Methods

### Preparation of fractions

The wild plant of *S. involucrata* used in this study was a gift from Biopure Biotechnology (Changhua, Taiwan). Twenty grams of dried aerial and flower parts *S. involucrate* was extracted with 100 mL of methanol three times under reflux for 2 h. The methanol extracts (SI-1) were combined, and the solvent was evaporated in vacuum to give a deep brown syrup. The syrup was resuspended in water and then partitioned successively with pentane, ethyl acetate (SI-2) and n-butanol (SI-3) to leave a water layer (SI-4). The solvents were evaporated respectively, and the residues were used throughout this study.

### Reverse-phase high-performance liquid chromatography (HPLC) analysis of flavonoids in S. involucrata

The determination of flavonoids from *S. involucrata* was carried out by HPLC with a photodiary detector. The HPLC system consisted of a Shimadzu LC-20AT solvent delivery system, equipped with a SPD-M20A photodiode array detector, set at 270 nm. Samples were injected with SiL-20A autosample to separate on the TSK-Gel ODS-100S column. The column was maintained at an ambient temperature of 25°C. The flow rate of the system was 1.0 mL/min. The mobile phase consisted of solvent A (0.3% formic acid) and solvent B (acetonitrile). The elution profile for A was 0-10 min, with a linear gradient change of 0-5%; 10-40 min, with a linear gradient change to 55%; and maintained for another 10 min with a post run time to equilibrate the column and for the baseline to return to the normal and initial working conditions [2].

### Chemicals and reagents

3,4-dihydroxyphenylacetic acid (DHPAA), 3,4-dihydroxytoluene (DHT), 3-hydroxyphenylacetic acid (HPAA) and 4-hydroxy-3-methoxyphenylacetic acid (homovanillic acid, HVA) and 3-(4,5-Dimethylthiazol-2-yl)-2,5-diphenyl tetrazolium bromide (MTT) were purchased from Sigma (St. Louis, MO). LPS (from Escherichia coli 0111:B4), mouse antibodies against mouse phospho-ERK1/2, phospho-JNK1/2, phospho-p38, and actin were purchased from Sigma (St. Louis, MO). Rabbit antibodies against mouse ERK1/2, JNK1, p38, phospho-IκB-α, iNOS, COX-2, and HRP-second antibodies were obtained from Santa Cruz Biotechnology (Santa Cruz, CA). IL-1β, IL-6, and TNF-α ELISA kits were purchased from R&D Systems (Minneapolis, MN).

### Cell culture

RAW 264.7 macrophages were obtained from the American Type Culture Collection (Rockville, MD). The cells were propagated in RPMI-1640 medium (Gibco Laboratories, Grand Island, NY) supplemented with 10% heat-inactivated fetal calf serum (Biological Industries Ltd, Kibbutz Beit Haemek, Israel), and 2 mM L-glutamine (Life Technologies, Carlsbad, CA) at 37°C in a 5% CO2 incubator.

### Cell viability assays

Cell viability assay was performed by the MTT (3-[4,5-dimethylthiazol-2-yl]-2,5 diphenyl tetrazolium bromide) assay based on the conversion of MTT into formazan crystals by living cells, which determines mitochondrial activity. Cell was cultured in 96-well plate with 5000/well confluence. Twenty-four hours after treatment, after removing supernatant 100 μl MTT reagent was added for 1 h incubation. For measurement, 100 μl DMSO was added in each well follow by ELISA reader detection at 570 nm. Data are representative of three independent experiments.

### Enzyme-linked immunosorbent assay (ELISA)

ELISA was performed according to the previous study [30]. Briefly, 2 × 10^6^ in 2 mL medium were seeded in 60 mm dishes for treatment. Six hours after treatment, 50 μL of biotinylated antibody and 50 μL of supernatant were added to a stripwell plate precoated with antimouse IL-1β, IL-6, and TNF-α antibodies and incubated at room temperature for 2 h. After 3 washes with washing buffer, 100 μL of diluted HRP-conjugated streptavidin concentrate was added to each well and the plate incubated at room temperature for 30 min. The washing process was repeated, then 100 μL of a premixed tetramethylbenzidine substrate solution was added to each well and the reaction developed at room temperature in the dark for 30 min. Following addition of 100 μL of stop solution to each well, the absorbance was read in a microplate reader at 450 nm. Data are representative of three independent experiments.

### NO measurement

1×10^5^ cells was seeded into 24-well plate (Nunc, USA) at the day before treatment. Twenty-four hours after treatment, the supernatant was collected and NO measurement was performed by Griess Reagent. Briefly, the supernatant (100 μl) was reacted with 1% sulfanilamide solution (50 μl) for 5 min in the dark. After incubation, 50 μl 0.1% NED solution was added and absorbance was measured at 550 nm. The concentration of NO was calculated by utilizing NaNO2 standard curve. Data are representative of three independent experiments.

### Western blot assay

After treatment, the cells were collected and lysed at 4°C in lysis buffer (25 mM Tris-HCl, pH 7.5, 100 mM NaCl, 2.5 mM EDTA, 2.5 mM EGTA, 20 mM NaF, 1 mM Na3VO4, 20 mM sodium β-glycerophosphate, 10 mM sodium pyrophosphate, 0.5% Triton X-100) containing protease inhibitor cocktail (Sigma, St. Louis, MO), then the whole cell lysate was separated by SDS-PAGE and electrotransferred to a PVDF membrane. The membranes were incubated for 1 h at room temperature in blocking solution (5% nonfat milk in phosphate buffered saline with 0.1% Tween 20), then incubated for 2 h at room temperature with specific primary antibody in blocking solution. After 3 washes in PBS with 0.1% Tween 20, the membrane was incubated for 1 h at room temperature with HRPconjugated secondary antibody in blocking buffer and developed using an enhanced chemiluminescence Western blot detection system.

### Statistical analysis

All values are given as means ± S. E. Data analysis involved one-way ANOVA with subsequent Scheffé test. * and ** indicate a significant difference at the level of p < 0.05 and P < 0.01, respectively, compared to LPS alone.

## Results and discussion

**Figure 1:**
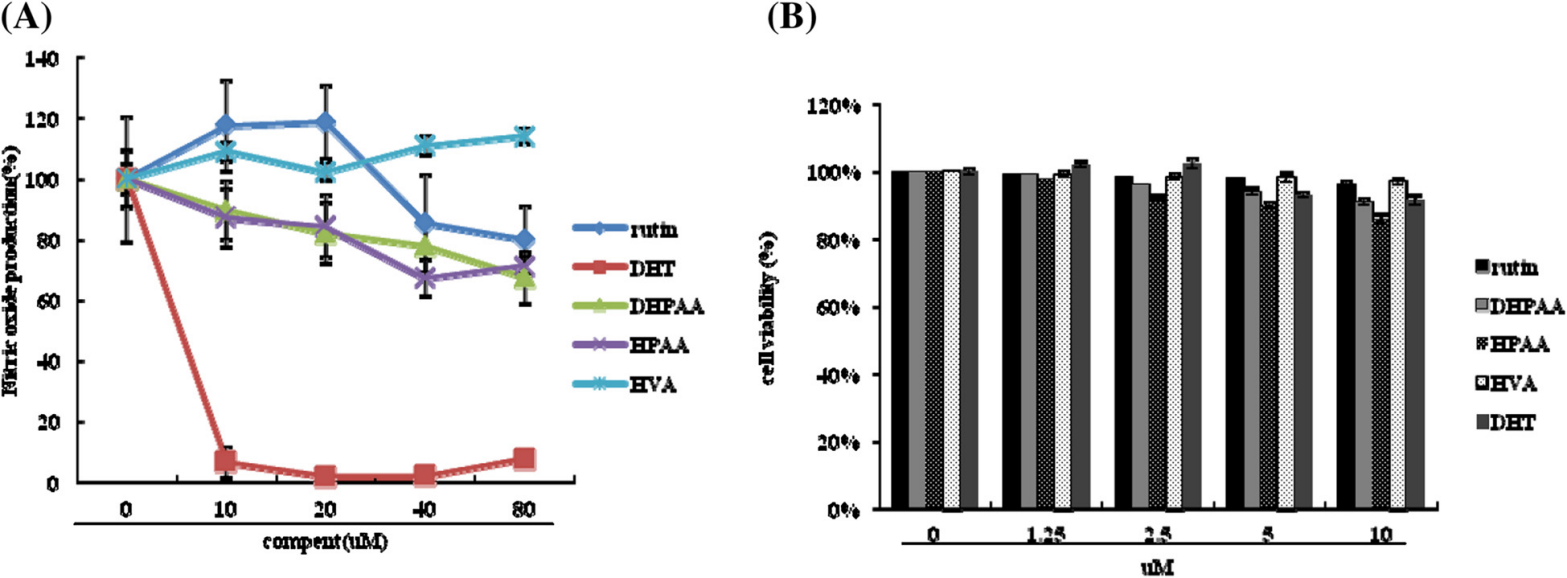
**Effects of rutin metabolites on NO production in LPS-stimulated RAW 264.7 and DHT cytotoxicity test. (A)** After LPS stimulation, RAW264.7 macrophage with rutin or its metabolites including DHT, DHPAA, HPAA and HVA treatment was assessed to NO production measurement. **(B)** Cell cytotoxicity assay for DHT. RAW 264.7 was treated with DHT for 24 hours, followed by incubating with MTT reagent to measure OD 570 nm by spectrophotometry. The data was expressed as the means ± SE from three separated experiments.

**Figure 2:**
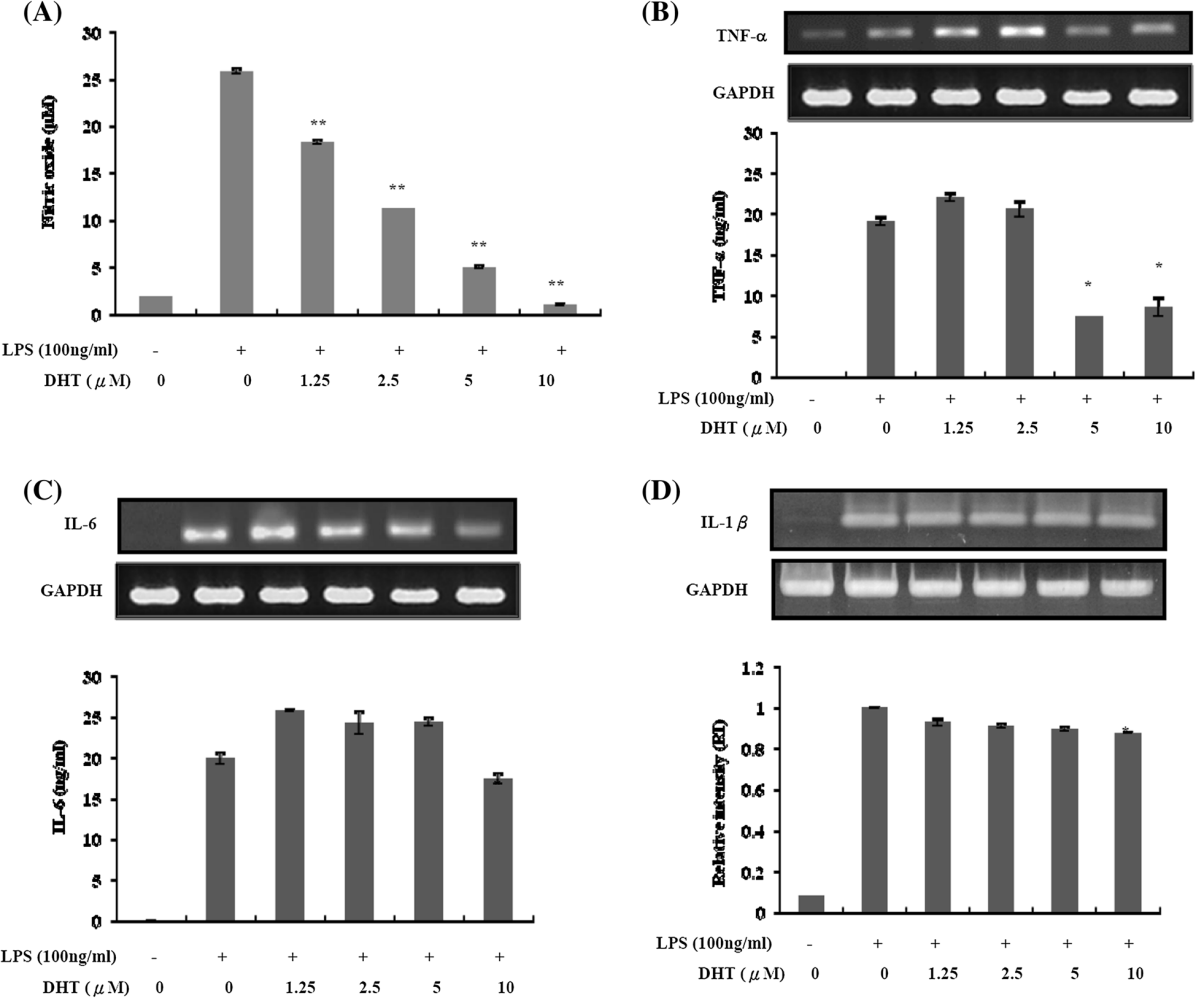
**Effect of DHT on inhibition nitric oxide and pro-inflammatory cytokines production in LPS-stimulated RAW264.7 macrophage.** RAW264.7 macrophage were pre-treated with DHT for 30 min, followed by stimulating with LPS (100 ng/ml) for 6 and 24 hours. **(A)** After incubation for 24 h, nitric oxide concentration in culture medium was assayed by the Gress reaction. After incubation for 6 h, the **(B)** TNF-α and **(C)** IL-6 mRNA expression and secreted concentration in culture medium was assayed by RT-PCR and ELISA. **(D)** The levels of IL-1β mRNAs and protein were determined by RT-PCR and ELISsA. The data was expressed as the means ± SE from three separated experiments. *P < 0.05, **P < 0.01, significantly with the LPS-treated group only.

To test whether rutin and its’ metabolites can efficiently inhibit inflammation, we measured nitric oxide (NO) production, an inflammation marker, in LPS-stimulated RAW 264.7 cells in the present of these compounds. The result indicated 10 μM DHT had the most significant inhibitory effect on NO production when compared with other metabolites (Figure 1A). Therefore, we further utilized DHT to address its biological effects and mechanisms on inflammation. In the mention of cytotoxicity, the cell viability was over 80% after 10 μM rutin or metabolites treatment suggested that these compounds had low cytotoxicity. (Figure 1B). To evaluate the effect of DHT on NO production precisely, we performed NO measurement for RAW264.7 cell with different dose. The result indicated that DHT can significantly reduce NO generation after LPS treatment in a dose-dependent manner from 1.25 to 10 μM (Figure 2A). In addition to NO, we also test several inflammatory cytokines production after LPS stimulation including TNF-α, IL-6 and IL-1β. DHT at 5 and 10 mM can significantly reduce the mRNA and protein expression levels of TNF-# (Figure 2B). No significant effect was observed on IL-6 and IL-1b mRNA and protein expression (Figure 2C and D). Taken together, these results indicated that DHT can eliminate LPS-induced NO and inflammatory cytokines production majorly through TNF-α signaling without affecting cell viability.

**Figure 3:**
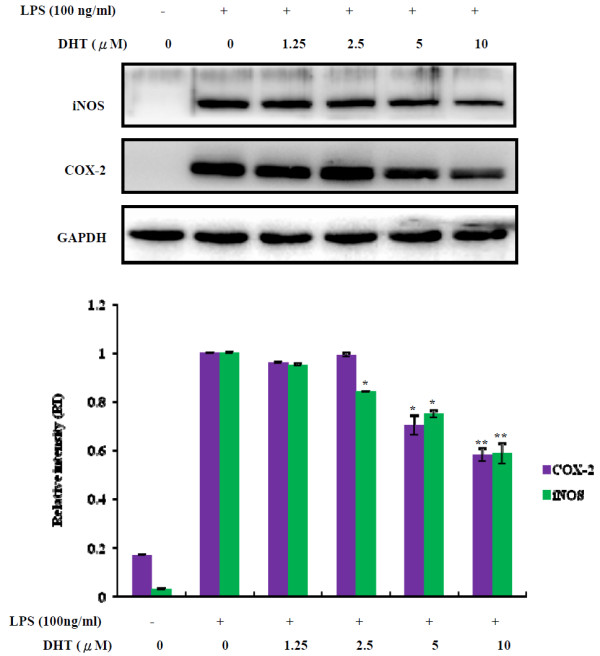
**Effects of DHT on inhibition of COX-2 and iNOS expression in LPS-stimulated RAW264.7 macrophage.** RAW264.7 macrophage were pre-treated with DHT for 30 min, followed by stimulating with LPS (100 ng/ml) for 24 hours. The protein levels of COX-2, iNOS and GAPDH were determined by Western blot analysis. (upper panel) RI (Relative intensity) was measured by the densitometry scanning (lower panel). The data was expressed as the means ± SE from three separated experiments. *P < 0.05, **P < 0.01, significantly with the LPS-treated group only.

**Figure 4:**
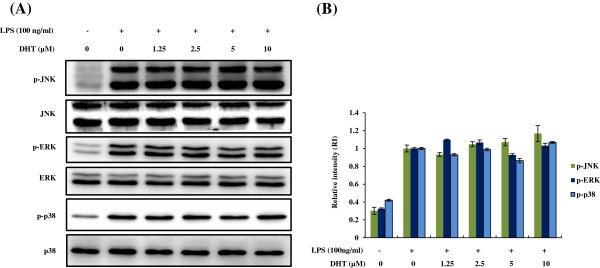
**Effects of DHT on activation of MAPK pathway in LPS-stimulated RAW264.7 macrophage.** RAW264.7 macrophage were pre-treated with DHT for 30 min, followed by stimulating with LPS (100 ng/ml) for 30 min. **(A)** The phosphorylation and total protein levels of MAPK (ERK1/2, JNK1/2 and p38) were determined by Western blot analysis. **(B)** Quantification of the level of phosphorylation measured by the densitometry scanning. The data was expressed as RI (relative intensity) and the means ± SE from three separated experiments. *P < 0.05, **P < 0.01, significantly with the LPS-treated group only.

Since NO production can be catalyzed by inducible NO synthase (iNOS), we further investigated the effect of DHT on iNOS expression. The result showed that DHT can significantly reduce iNOS expression in a dose dependent manner. Similar results were obtained for cyclooxygenase-2 (COX-2) (Figure 3). These results indicated that the DHT was the principal metabolite of rutin with potential anti-inflammatory activity. MAPK signaling was one of the most important LPS-activated pathways, therefore, we tested whether DHT can modulate LPS-stimulated downstream signaling of MAPK. The results indicated that DHT had no effect on LPS mediated JNK1/2, ERK1/2 as well as p38 signaling (Figure 4A-B).

**Figure 5:**
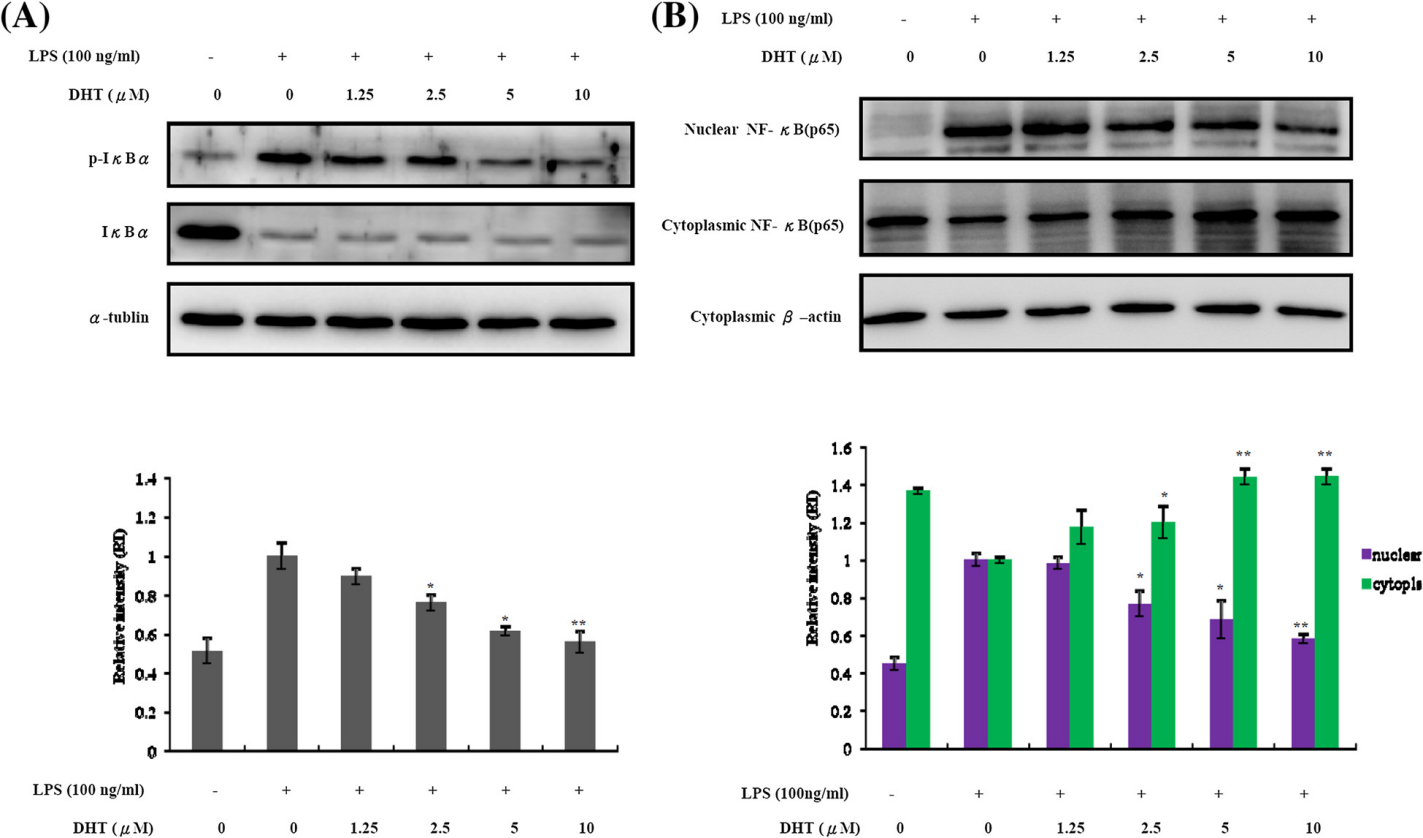
**Effect of DHT on activation of NF-kB pathway in LPS-stimulated RAW264.7 macrophage.** RAW264.7 macrophage were pre-treated with DHT for 30 min, followed by stimulating with LPS (100 ng/ml) for 30 min. **(A)** The phosphorylation and total protein levels of IκB and α-tubulin were determined by Western blot analysis. **(B)** The p65 subunit of NF-kB in cytoplasm and p65 in nucleus were determined by western blot analysis. RI (Relative intensity) was measured by the densitometry scanning. The data was expressed as the means ± SE from three separated experiments. *P < 0.05, **P < 0.01, significantly with the LPS-treated group only.

**Figure 6:**
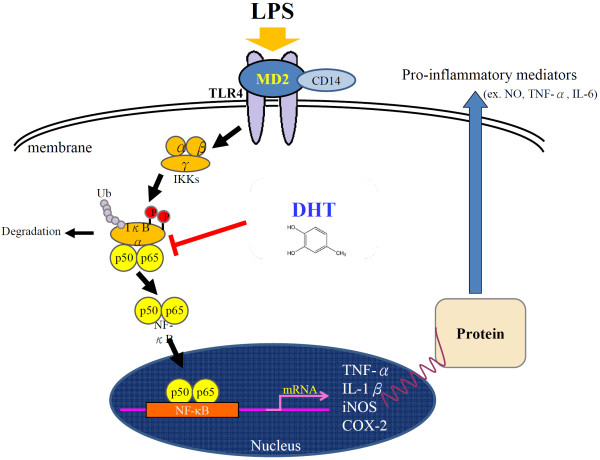
Illustration of signaling for RAW264.7 macrophage anti-inflammatory effect by DHT.

The NF-κB, one of the critical transcription factors, regulates the inflammatory-related gene expression in the LPS-activated macrophages. In the resting macrophages, NF-κB is sequestered in the cytoplasm as an inactive precursor complex by its inhibitory protein, IκB. After stimulation, IκB is phosphorylated by IκB kinase, ubiquitinated, and rapidly degraded through proteasomes followed by NF-κB releasing. Released p65 subunit of NF-κB is translocated from cytoplasm to nucleus and activates numerous corresponding genes. We found that DHT can significantly reduce the phosphorylation of IκB-a in RAW 264.7 cells after LPS treatment (Figure 5A). This leaded to accumulation of NF-κB (p65) in the cytoplasm and nuclear NF-κB (p65) reduction (Figure 5B). These results indicated that DHT at least can attenuate NF-κB signaling activation in macrophages after LPS treatment. Taken together, our results showed that DHT can efficiently inhibit IκB-NFκB mediated inflammatory response (Figure 6).

Inflammation not only participates in host defense but also controls the pathogenesis of numerous diseases including type 2 diabetes mellitus [31-34], atherosclerosis [35,36], obesity [31,36], silicosis [37], Alzheimer's disease [38], gout [33,39] and kidney disease [40,41]. An elevated level of circulating inflammatory cytokines, in particular, and a combined elevation of IL-1 and IL-6, constitute risk factors for the pathogenesis of type II diabetes mellitus [42]. Also, TNF-α was found in some metabolic diseases [43,44]. Some genetic engineering animal model further confirmed inflammatory progress had impact in vascular disease [45]. Therefore, reduction of inflammation may provide an alternative disease management in complementary medicine field.

In this study, we found that rutin and DHT, the phenol derivative of rutin, can significantly reduce NO production in inflammatory response of LPS-stimulated macrophages. Although NO played a critical role in the host defense on various pathogens, the overproduction of NO can be harmful and result in septic shock, rheumatoid arthritis, and autoimmune diseases [46]. Therefore, adjuvant therapeutic agents that inhibit iNOS might be useful in relieving these inflammatory conditions. In this study, DHT can potentially inhibit iNOS as well as COX-2 expression through its upstream signaling in LPS-stimulated macrophages. The dual intrinsic enzyme activities of COX-2 catalyze 2 sequential reactions in the metabolism of arachidonic acid (AA). In addition, the COX-2 enzyme possesses a heme-containing active site that provides peroxidase activity, which requires 2 electrons (2e*−*) to become active. The peroxidase reaction converts PGG2 to PGH2 by removing oxygen(s), (Ox), which might be a source of oxygen radicals. Therefore, as more AA is metabolized to PG by COX-2, more electron donors are depleted and more oxygen radicals are generated. The effect of DHT on COX-2 expression also supported its potential role and application in physiological inflammatory unbalance by health supplements.

The reduction of iNOS and COX-2 may be due to transcription suppression. Among transcription factors, NF-*κ*B is a significant inflammation-related one for downstream gene activation including cytokines, chemokines, adhesion molecules, and acute phase proteins [47]. In addition, NF-*κ*B is a redox-sensitive transcription factor that regulates a multitude of inflammatory genes, including cytokines, chemokines, adhesion molecules, and acute phase proteins. Under basal conditions, NF-*κ*B is inactive by associated with inhibitory proteins in the cytoplasm to prevent DNA binding for transcription activity. In our study, after the treatment with DHT, the nucleic NF-*κ*B p65 was reduced while cytoplasmic one was accumulated (Figure 5). Although the reduction of iNOS and COX-2 may be the consequence of NF-*κ*B inactivation, other possible mechanisms involved in DHT effects can’t be excluded due to wide contribution of NF-*κ*B. Therefore, DHT may affect the upstream molecular of I*κ*B-NF-*κ*B signaling, since the phosphorylation of I*κ*B was attenuated by DHT. To clarify the underlying molecular networks, more experiments should be further performed. On the other hand, in this study, we found DHT had no influence on MAPK pathway (Figure 4) suggested DHT reduced TNF-α expression majorly through PI3K/AKT followed by NF-*κ*B activation.

## Conclusion

We demonstrated the anti-inflammatory activity of DHT, the phenol derivative of rutin, via affecting I*κ*B-NF-*κ*B signaling in macrophages and dissected its possible mechanism. The anti-inflammatory activity might be a basis for therapeutic supplements innovation in complementary and alternative medicine.

## Competing interests

The authors declare that they have no competing interests.

## Authors’ contributions

YLC made contributions to conception, design and gave final approval of the manuscript to be published. YPC performed experiments and collected the data for analysis. KFH and KYS provided the interpretation of data and were involved in drafting the manuscript. All authors read and approved the final manuscript.

## Pre-publication history

The pre-publication history for this paper can be accessed here:

http://www.biomedcentral.com/1472-6882/14/21/prepub
